# Single cell RNA-Seq reveals pre-cDCs fate determined by transcription factor combinatorial dose

**DOI:** 10.1186/s12860-019-0199-y

**Published:** 2019-06-28

**Authors:** Wenji Ma, Jaeyop Lee, Daniel Backenroth, Yu Jerry Zhou, Erin Bush, Peter Sims, Kang Liu, Yufeng Shen

**Affiliations:** 1 Department of Systems Biology, Columbia University Medical Center, New York, NY 10032 USA; 2 Department of Microbiology and Immunology, Columbia University Medical Center, New York, NY 10032 USA; 3 JP Sulzberger Columbia Genome Center, Columbia University Medical Center, New York, NY 10032 USA; 4 Department of Biomedical Informatics, Columbia University Medical Center, New York, NY 10032 USA; 5 Current Address: Boehringer Ingelheim Pharmaceutical Inc., Ridgefield, CT 06877 USA

**Keywords:** Dendritic cell, Cell differentiation, Transcriptional factor, Single cell RNA-Seq

## Abstract

**Background:**

Classic dendritic cells (cDCs) play a central role in the immune system by processing and presenting antigens to activate T cells, and consist of two major subsets: CD141^+^ cDC (cDC1) and CD1c^+^ cDC (cDC2). A population of migratory precursor cells, the pre-cDCs, is the immediate precursors to both cDC subsets. Previous studies showed that there were two pre-committed pre-cDC subpopulations. However, the key molecular drivers of pre-commitment in human pre-cDCs were not investigated.

**Results:**

To identify the key molecular drivers for pre-commitment in human pre-cDCs, we performed single cell RNA sequencing (RNA-Seq) of two cDC subsets and pre-cDCs, and bulk RNA-Seq of pre-cDCs and cDCs from human peripheral blood. We found that pre-DC subpopulations cannot be separated by either variable genes within pre-cDCs or differentially expressed genes between cDC1 and cDC2. In contrast, they were separated by 16 transcription factors that are themselves differentially expressed or have regulated targets enriched in the differentially expressed genes between bulk cDC1 and cDC2, with one subpopulation close to cDC1 and the other close to cDC2. More importantly, these two pre-cDC sub-populations are correlated with ratio of *IRF8* to *IRF4* expression level more than their individual expression level. We also verified these findings using three recently published datasets.

**Conclusions:**

In this study, we demonstrate that single cell transcriptome profiling can reveal pre-cDCs differentiation map, and our results suggest the concept that combinatorial dose of transcription factors determines cell differentiation fate.

**Electronic supplementary material:**

The online version of this article (10.1186/s12860-019-0199-y) contains supplementary material, which is available to authorized users.

## Background

The hematopoietic system is one of the best model systems for the study of lineage differentiation and diversification. One type of hematopoietic cells, dendritic cells (DCs), plays a central regulatory role in the immune system. It detects pathogen-associated molecular pattern (PAMP) signal or danger-associated molecular pattern (DAMP) signal, captures antigens from self or invaders, processes and presents them to activate T cells, and sustains the memory adaptive immune response and Tregs, thereby bridging innate and adaptive immune response [1]. The functional complexity of DCs is reflected in their diversity. Conventional dendritic cells (cDCs) in humans that activate T cells are comprised of two subsets with distinct phenotypes and functions, namely, CD141^+^ cDCs (cDC1) and CD1c^+^ cDCs (cDC2). Specifically, human cDC1 and their equivalent in mouse, CD8^+^ cDCs, specialize in activating CD8 T cell response [2], whereas human cDC2 and their murine counterpart CD11b^+^ cDCs, specialize in activating CD4 T cell response [3]. Both cDC1 and cDC2 descend from migratory dendritic cell precursor cells, or pre-cDCs, which arise from common dendritic cell progenitor (CDP) in the bone marrow and egress to the periphery where they terminally differentiate into cDCs [4]. Several phenotypically distinct pre-cDC cell populations have been identified in human peripheral blood with potential to produce cDC1 and cDC2 [5–8].

Single cell RNA sequencing (scRNA-Seq) studies not only led to the identification of a new pre-cDC population in human blood [7], but also revealed that seemingly homogeneous pre-cDCs in mouse and human were heterogeneous and led to the discovery that pre-cDCs have two subpopulations pre-committed to cDC1 and cDC2, respectively [5, 6, 9]. However, the identification and characterization of the pre-cDC subpopulations in these studies were not based on scRNA-Seq data, but mainly from prospective isolation of cells, followed by bulk RNA-Seq. It remains unclear whether we can use scRNA-Seq data directly to separate and identify pre-committed subpopulations. Additionally, the transcriptional program that drives pre-cDC pre-commitment is not well characterized.

A number of important transcriptional factors have been shown critical in cDC development in general. cDC1 dependent transcriptional factors include *IRF8* [10], *ID2* [11], *BATF3* [10, 12], *NFIL3* [13]. cDC2 dependent transcriptional factors include *IRF4* [14, 15], *KLF4* [16], *ZEB2* [17]. There are some transcription factors implicated in both cDC1 and cDC2 development, such as Ets-family transcriptional factor *PU.1* (*SPI1*), *ZBTB46*, *E2–2* (*TCF4*), *STATs*, *IKZF1* (*IKAROS*) and *Notch RBP-J* [18]. However, the recipes of essential transcription factors for cDC1 and cDC2 subset commitment in human pre-cDCs have not been investigated. Our recent studies indicate that the lineage program is established early in hematopoietic stem cells (HSCs) and multipotent progenitors (MPPs) and transmitted to progeny and strengthened during cell division, and suggest that the lineage program is correlated with and orchestrated by combinatorial dose of multiple transcription factors [19]. How to identify such lineage program recipes as a commonality shared between distinct development stages has not yet been established.

Here we report a single cell transcriptomics study of pre-cDCs to investigate core transcriptional program underlying cDC1 and cDC2 lineages. We used the Fluidigm C1 platform to sequence mixed pre-cDCs, cDC1, and cDC2 cells, and developed a computational approach to find master regulator transcription factors that drive the pre-cDC differentiation process. We ask 1) whether single-cell transcriptome profiling can distinguish two cDC subsets and their immediate precursor pre-cDCs; 2) whether pre-cDCs are composed of distinct committed subpopulations that resemble terminally differentiated cDC cells; and 3) which transcription factors drive the terminal differentiation of pre-cDCs. To answer these questions, we implemented a workflow as shown in Additional file 1: Figure S1 Specifically, we used multidimensional scaling (MDS) to infer cell types based on scRNA-Seq data and remove outliers. We then identified genes with highly variable expression in pre-cDC population and showed that they can be used to separate cDC1 and cDC2, indicating the heterogeneity of pre-cDCs is associated with cDC commitment. Finally, we identified transcription factors that are differentially expressed between bulk cDC1 and cDC2 (indicated in red and blue) or have targets enriched among differentially expressed genes (indicated in olive), and showed that these are potential master regulators that underline pre-cDC heterogeneity and drive differentiation.

## Results

### Single cell global transcriptome can identify human pre-cDCs, cDC1s and cDC2s from mixture independent of surface marker

**Fig. 1:**
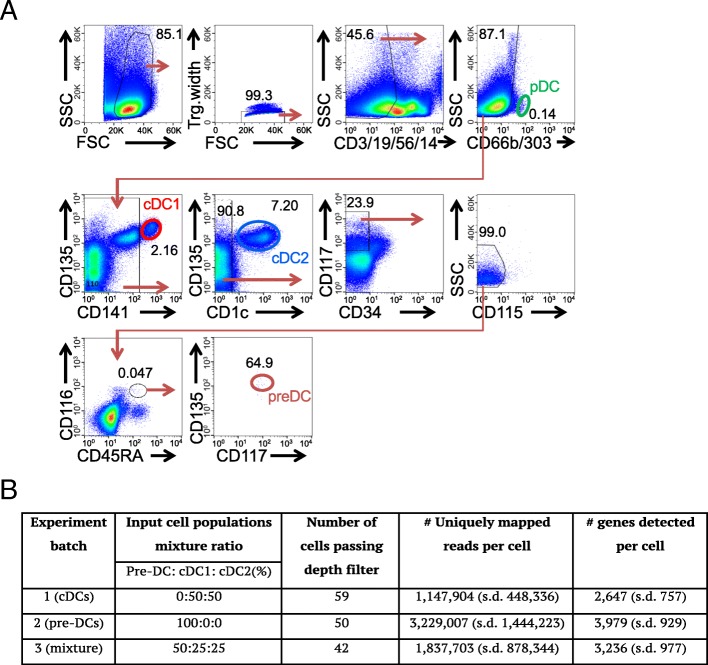
Experiment design and sample summary. **a** Gating strategy for isolating pre-cDCs and cDC1s and cDC2s. **b** Sequencing batch experiments details

To determine whether single cell transcriptome can be compared across different experiments, and can distinguish pre-cDC, cDC1 and cDC2 from cell mixture without prior knowledge of cell identity, we isolated pre-cDC, cDC1 and cDC2 from peripheral blood as in Fig. 1a, and mixed them with different combinations in three batches and submitted each batch for single cell RNA-Seq using the Fluidigm C1 system. The pre-DCs here representing 0.001% of CD45^+^ PBMC are Lin^−^CD45RA^+^CD115^−^CD116^+^CD117^+^Flt3^+^, distinct from pDC in cell surface expression of CD303 (Fig. 1a) and transcriptome (Additional file 1: Figure S2A) and lack pDC potential [4]. They are phenotypically different from Lin^−^CD33^+^CD45RA^+^CD123^+^ pre-DCs from the work of See et al. [6] and CD100^hi^CD34^int^FLT3^−^ Villani et al. [7] (Additional file 1: Figure S2B).

**Fig. 2:**
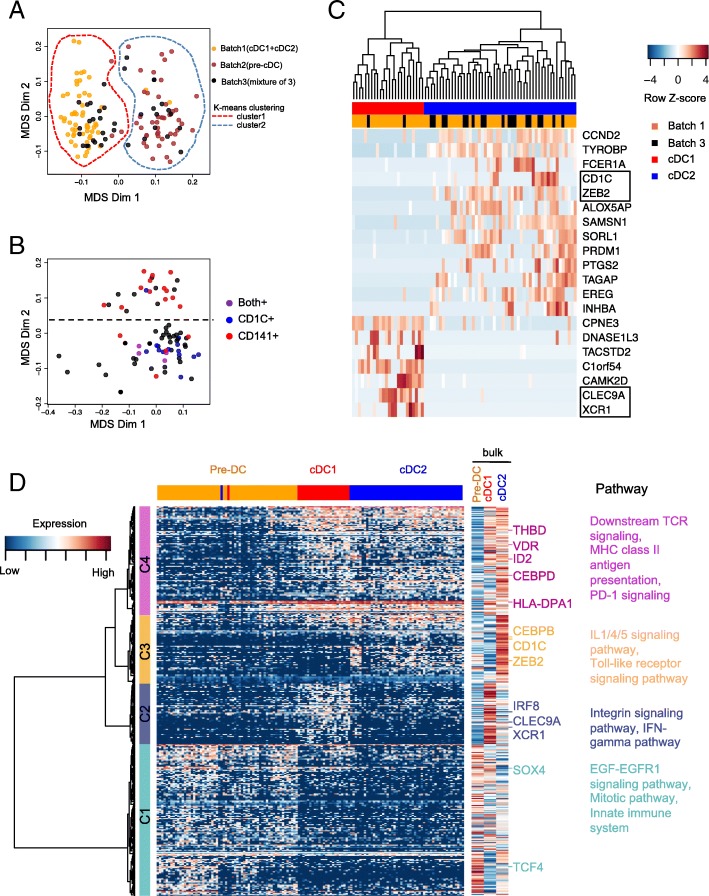
Cell type identification of the single cells from mixture with MDS based on global transcriptome. **a** Multidimensional scaling (MDS) plot for the cells that passed quality control, batch1 (yellow, cDCs), batch2 (brown, pre-cDCs) and batch3 (black, mixture of cDCs and pre-cDCs), where the input distance was adjusted for dropout rate with SCDE. The cells can be separated into 2 clusters by k-means clustering: cluster1 for cDCs and cluster 2 for pre-cDCs. **b** The clustering pattern of cluster1 identified in panel **a** with MDS. Each cell is colored with binary expression of CD141(red), CD1c (blue), both (purple) or none (black). **c** Heatmap of the top 20 differentially expressed genes derived by SCDE by comparing the upper and lower groups of cDCs in panel **b**. The top dendrogram was generated with hierarchical clustering. The two column color bars represent batch and cell type information, respectively. Genes surround by black box are known key genes for cDC differentiation. **d** Transcriptional signature of pre-cDC, cDC1 and cDC2 population. Three hundred eighty genes shown in rows were differentially expressed in at least two populations of pre-cDC, cDC1 and cDC2 computed with ANOVA (false-discovery rate (FDR) < 0.05) and can be classified into 4 clusters(C1–4) indicated by the row slide color. Bulk expression of the three cell types for these genes after averaging over 3 biological replicates is also shown on the right. Right margin also shows the predicted upstream regulators and enriched pathways for each gene cluster. Single cells in the columns were ordered by the hierarchical clustering result based on the 380 genes

To test whether scRNA-Seq data can separate DCs and pre-DCs, we mixed cDC1, cDC2 and pre-DCs prior to sequencing. Specifically, in our experiment design, batch 1 contained cDC1:cDC2 in a ratio of 1:1, batch 2 contained only pre-cDC, and batch 3 contained pre-cDC: cDC1: cDC2 in a ratio of 2:1:1. The cells that passed a *depth filter* of 100,000 reads mapped to gene region comprise 59, 50 and 42 cells from batch 1, batch 2 and batch 3, respectively (more details in Fig. 1b and quality control data in Additional file 1: Figure S3A-B). With these cells, we first calculated the pair-wise transcriptome distance using the global transcriptome of each cell (see Methods), and generated a 2-dimensionsional MDS plot of the distance matrix to visualize the transcriptomic similarities between the cells (Additional file 1: Figure S4A). The resulted plot revealed two groups of cells that are densely clustered, and a third group of cells sparsely scattered after performing k-means clustering on the MDS plot. These sparsely scattering cells in group 3 have significantly lower read depths than the other cells (Additional file 1: Figure S4B), and are thus removed from the downstream analysis as technical outliers. We further filtered the cells by mitochondrial reads percent < 30% and number of expressed genes > 1000, leading to 135 single cells. These single cells express most of the top 100 housekeeping genes [20] and express few of the cell cycle genes (Reactome, http://www.reactome.org), indicating that they are alive and not undergoing cell cycle (Additional file 1: Figure S4C). Among the 135 single cells with good RNA-Seq data quality, 52/54 cells from batch 1 (only containing cDC1 and cDC2) are clustered in cluster1, suggesting its identity of *cDC cluster*; 44/46 cells from batch 2 (only containing pre-cDCs) are clustered in cluster2 suggesting its identity of *pre-cDCs cluster*. Importantly, 19 and 15 cells in batch 3 are distributed in and blended with cluster1 (cDCs) and cluster2 (pre-cDCs), respectively (Fig. 2a), consistent with its cellular composition (cDC1:cDC2:pre-cDC, 1:1:2). This indicates that the batch effect is negligible so that cells can be compared cross experiments. We conclude that global transcriptome can separate cDCs from pre-cDCs without known identity.

We then ask whether single cell global transcriptome could distinguish cDC1 and cDC2. Using MDS, we further separated the 78 cells in group 1, i.e., putative cDCs, into two distinct clusters, which are correlated with cDC1 and cDC2, respectively, based on the expression of *CD141* and *CD1c* transcripts (Fig. 2b**)**. Using SCDE [21], we identified top 20 differentially expressed genes ranked by absolute value of Z-score between the two visible clusters of cDCs (Fig. 2c). The upper group of cells in Fig. 2b highly express *CLEC9A* and *XCR1*, indicating its identify of cDC1, whereas the lower group of cells in Fig. 1b highly express *CD1C* and *ZEB2*, indicating its identity of cDC2 (Fig. 2c). cDC cells from both batch 1 and batch 3 are distributed in two clusters (Fig. 2c). Additionally, signature analysis indicates the high consistency of cell type identity in the two clusters of single cells (Additional file 1: Figure S5). These results indicate that global transcriptome can separate cDC1 from cDC2 without known identity.

To further verify the pre-cDC, cDC1 and cDC2 populations inferred from their global transcriptome with MDS, we identified 380 differentially expressed genes between at least one pair of cell populations in mean expression with one-way analysis of variance (ANOVA) under the criteria: absolute value of log_2_ fold change > 1 and false-discovery rate (FDR) < 0.05. Hierarchical clustering of these differentially expressed genes on all the good single cells revealed 4 major clusters of genes (Fig. 2d**,** Additional file 2: Table S1). We used ConsensusPathDB-human(CPDB) [22], an aggregation tool that integrate pathways from different sources for human, to infer pathways enriched within each gene cluster. Cluster 1 genes were highly expressed in pre-cDCs and low in cDCs, including key genes like *SOX4* and *TCF4,* enriched for development pathways like EGF-EGFR1 signaling pathway and mitotic pathway*.* Cluster 2 genes were highly expressed in cDC1, including *CLEC9A*, *XCR1* and transcriptional factor *IRF8,* enriched for integrin signaling pathway and IFN-gamma pathway. Cluster 3 genes were highly expressed in cDC2s, including the surface marker *CD1c* and transcription factor *CEBPB and ZEB2,* enriched for IL1/4/5 signaling pathway and Toll-like receptor signaling pathway. Cluster 4 genes were highly expressed in both cDC1s and cDC2s, including *VDR*, *CEBPD* and HLA-D* genes, enriched for downstream TCR signaling, MHC class II antigen presentation and PD-1 signaling. Importantly, the expression pattern of these 380 genes was highly consistent between single cells and bulk populations (Fig. 2d), which confirms our inferred identities of the single cells from mixture and the good quality of our single cell data.

### Genes highly variable in pre-cDCs are associated with cDC specification

**Fig. 3:**
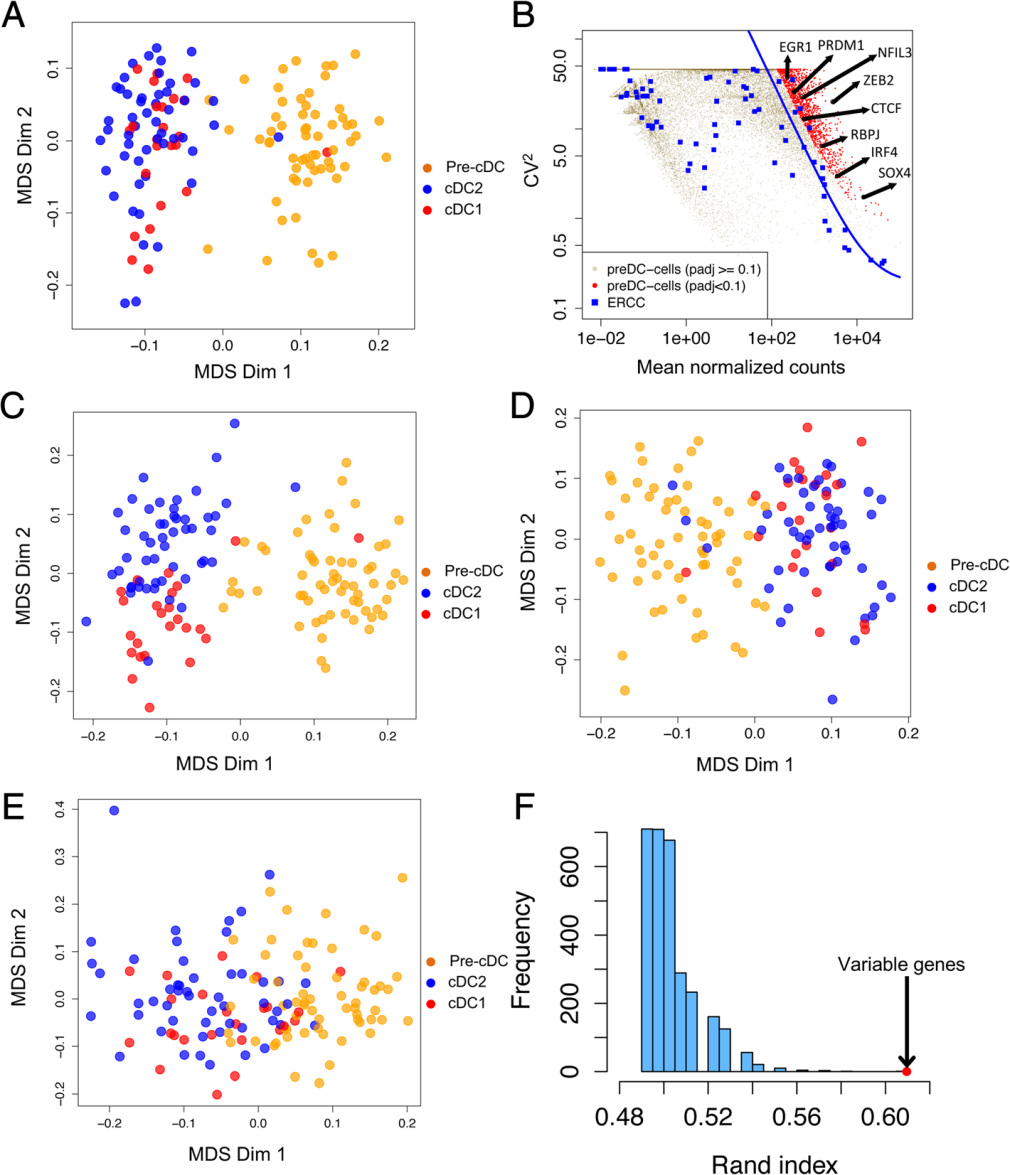
Highly variable genes in pre-cDCs population can separate DC subsets. **a** MDS plot of all the single cells based on global transcriptome. **b** Squared coefficient of variation (CV^2^) was plotted against the mean of normalized read counts for each gene of 50 pre-cDCs in Batch 2. The solid blue curve denotes the fitted variance-mean dependence with ERCC spike-ins. The genes marked in red show higher expression variability than background/technical noise (measured with spike-ins, blue) by testing the null hypothesis that the coefficient of biological variation is less than 50% with FDR < 0.1. **c** MDS plot of all the single cells with the 842 biologically variable genes. **d** MDS plot of all the single cells with randomly 842 sampled genes without replacement from genes that have mean expression > 100 in panel A. **e** MDS plot of all the single cells with 496 cell cycle genes downloaded from Reactome (http://www.reactome.org). **f** Histogram of rand index between the clustering result based on 842 sampled genes and the inferred cDC identities in Fig. 2. Larger rand index indicates higher consistence between two clustering results. Genes of high expression were randomly selected for 3000 rounds. The red dot indicates the rand index between the DC clusters based on the 842 variable genes and the inferred cDC identities

Recent studies have shown that pre-cDCs in mouse [9] and human [5] contain two distinct populations pre-committed to cDC1 and cDC2. We asked whether global transcriptome was able to separate pre-cDCs, using MDS. However, although global transcriptome could separate pre-DCs from cDCs, it was unable to separate pre-cDCs into two obvious subpopulations (Fig. 3a). We then set out to identify a subset of key genes that are associated with cDC specification in pre-cDCs. We hypothesize that if individual pre-cDCs are pre-committed to one of the two cDC lineages, the key genes should demonstrate more expression variability across individual pre-cDCs than background noise. We first estimated background noise with spike-ins, and then identified 842 variable genes that are 1) significantly more variable than background model (FDR < 0.1) and 2) exhibiting detectable mRNA (aligned reads number > 0) in at least 30 single cells (Fig. 3b and Additional file 2: Table S2). With MDS, expression of these 842 variable genes identified and separated pre-cDC, cDC1 and cDC2 cells (Fig. 3c), with separation of cDC1 and cDC2 better than that using global transcriptome (Fig. 3a). By contrast, both a random selection of 842 genes that have high mean expression in pre-cDCs (Fig. 3d**,** Additional file 2: Table S3), and cell cycle genes (Fig. 3e**,** Additional file 2: Table S4) failed to separate cDC1 and cDC2. To strength the conclusion from random selected genes, we randomly selected 842 genes with high expression for 3000 rounds. We used rand index to measure the similarity of the clustering result based on the randomly selected genes and the inferred identities of DCs from Fig. 2. Figure 3f shows that clustering result from variable genes is much better than that from randomly selected genes, indicating the best distinguishing ability of variable genes in pre-cDCs (Fig. 3f). This suggests that the key genes associating with cDC specification are already expressed and demonstrate high variability among individual pre-cDCs. Of the 842 variable genes, transcription factors include *ZEB2*, *IRF4* and *NFIL3* known for cDC differentiation and *CTCF* and *RBPJ* known for chromatin state remodeling (Fig. 3b). CPDB pathway analysis indicated enrichment in TGF-beta signaling pathway(Wikipathways), integrin (INOH) and interferon signaling(Reactome), and predicted *SPI1*, *TCF7*, *VDR*, *CIITA* and *IRF8 as* upstream regulators (Enrichr) which highly connect with dendritic cell development [23, 24] (Additional file 2: Table S2).

### Differentially expressed TFs between DC subsets can reveal pre-cDC differentiation trajectory

**Fig. 4:**
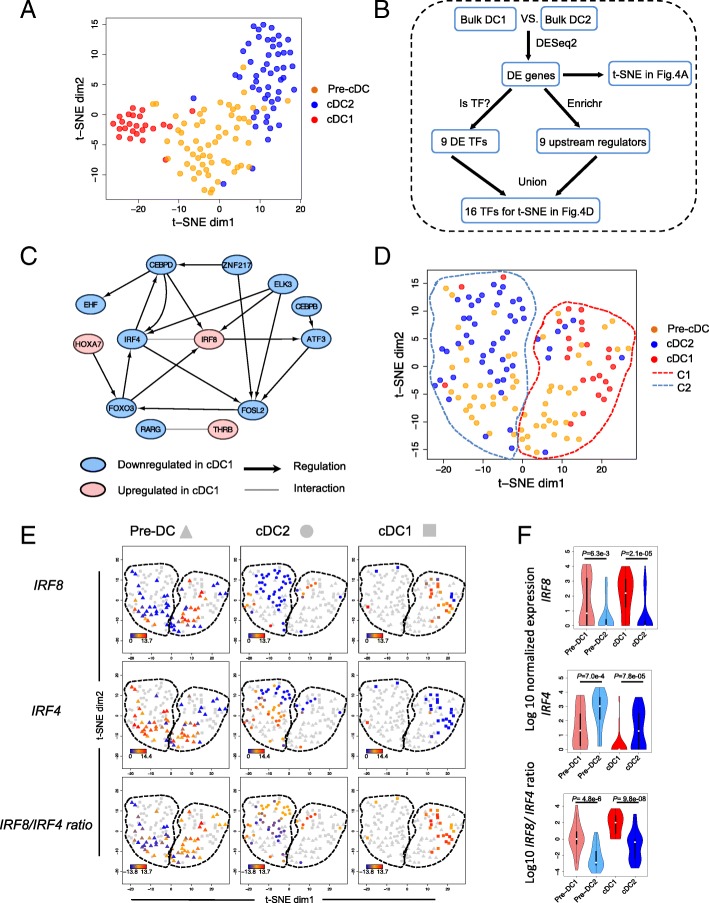
Pre-cDC subpopulation pattern in human is implicated by differentially expressed TFs between cDC1 and cDC2. **a** t-SNE plot of all the single cells with the full set of differentially expressed genes between bulk cDC1 and cDC2. **b** Flowchart of how to identify the MR TFs. **c** Regulatory network of the 13 out of 16 MR TFs based on the database of transcription factor and target relationship in human hematopoietic lineages from Neph et al. [25] complemented by String database [26]. **d** t-SNE plot of all the single cells with MR TFs that are differentially expressed between bulk cDC1 and cDC2. The cells were clustered into two groups C1 and C2 by k-means clustering. **e** Expression level of *IRF8*, *IRF4* and *IRF8/IRF4* expression ratio for each single cell. The three rows represent *IRF8*, *IRF4* and *IRF8/IRF4*, respectively. The columns represent pre-cDC, cDC2 and cDC1, respectively. For example, the *IRF8* expression level from low to high in pre-cDCs is represented from blue, orange to red, and the other cDC single cells are colored in gray. **f** Violin plot of *IRF8* expression*, IRF4* expression and *IRF8/IRF4* expression ratio in single cell populations: pre-cDC1, pre-cDC2, cDC1 and cDC2. *P* values indicated between pre-DC1 and pre-DC2 and between cDC1 and cDC2 are from Wilcoxon Rank Sum test

Since global transcriptome and variable genes within pre-DC populations fail to reveal the heterogeneity of pre-cDCs, we hypothesize that genes representing the lineage determinant of two cDC subsets should be able to identify two pre-cDC subpopulations that demonstrate consistency with cDC1 and cDC2, respectively. We first derived 234 differentially expressed genes (BH p.adj < 0.05, absolute value of log2 fold change > 1) between bulk cDC1 and cDC2 as DC signature gene set. As expected, this gene list successfully separated the single cDC1s from single cDC2s, however, it failed to reveal subpopulations in single pre-cDC cells shown in the t-Distributed Stochastic Neighbor Embedding (t-SNE) map (Fig. 4a), indicating that signature genes of differentiated cDCs are not yet fully manifested in pre-cDCs.

Given that the differentially expressed genes between cDC1 and cDC2 cannot identify subpopulations of pre-cDCs, we then asked whether the TFs that regulate the differentially expressed genes and drive the differentiation in the pre-cDCs can identify the pre-cDC subpopulations. Using a procedure as shown in Fig. 4b, we inferred a list of 16 candidate master regulator (“MR”) TFs: *ATF3, CEBPB, CEBPD, EHF, ELK3, FOSL2, FOXO3, HOXA7, IRF4, IRF8, JARID2, RARG, TCF7L2, THRB, ZEB2* and *ZNF217* that either: (a) are significantly differentially expressed between bulk cDC1 and cDC2 (BH p.adj < 0.05, absolute value of log2 fold change > 1, Additional file 2: Table S5); or (b) regulate the differentially expressed genes and exhibit differential expression between bulk cDC1 and cDC2 with marginal significance (*p*-value < 0.05, absolute value of log2 fold change > 1, Additional file 2: Table S5). Thirteen out of the 16 MR TFs form a network of genetic regulation and protein interaction (Fig. 4c**),** when analyzed using String database [26] and transcription factor and target database in human hematopoietic lineages from Neph et al. [25]. Expression level of these MR TFs demonstrated reasonable variability among pre-cDC, cDC1 and cDC2 in bulk and single cells (Additional file 1: Figure S6A-B). Using these MR TFs as input to t-SNE, pre-cDCs were separated into two distinct groups (Fig. 4d) that closely clustered with cDC1 cells and cDC2 cells, respectively. Here, dim1 is about the lineage divergence of pre-cDCs and dim2 is about the increasing commitment to cDC1/cDC2, and IRF4 and IRF8 have the highest correlation with both dim1 and dim2. We refer the pre-cDC subpopulation closer to the cDC1 cells as pre-cDC1, and the other pre-cDC subpopulation closer to the cDC2 single cells as pre-cDC2 (Fig. 4d). In contrast, neither global transcriptome, highly variable genes in pre-cDCs, or 234 differentially expressed genes between DC subsets was able to reveal subpopulation of pre-cDCs by tSNE visualization (Additional file 1: Figure S6C). To verify the separation power of the MR TFs, we performed a pseudo time trajectory analysis with Monocle2 [27]. In the resulting trajectory map, pre-DCs demonstrated differentiation branches with one branch committed to cDC1s, the other branch committed to cDC2s and a third group of likely less committed pre-cDCs at the bottom (Additional file 1: Figure S7A). For the sake of comparing this trajectory with t-SNE result, we flipped the two branches as shown in Additional file 1: Figure S7B. The pre-cDCs close to cDC1, cDC2 and those less committed at the bottom were encoded with boxes, triangles and asterisks, respectively. If we map this shape encoding into t-SNE plot, we can see most of the asterisks locate at the bottom, boxes locate at right and triangles locate at left (Additional file 1: Figure S7C), which means Monocle2, a very different analysis method confirms our finding from t-SNE. In contrast, Monocle2 analysis using variable genes failed to reveal trajectory of pre-DCs (Additional file 1: Figure S7D). This indicates that the candidate MR TFs, but not differentially expressed genes, variable genes or global transcriptome, are effective in separating the pre-cDC subpopulations.

Among the 16 MR TFs, *IRF8* and *IRF4* were known to be specific to cDC1s and cDC2s, respectively [10, 14, 15]. Indeed, our single RNAseq data indicates that expression of *IRF8* was high in most cDC1s and low in most cDC2s (Fig. 4e) whereas expression of *IRF4* was low in most cDC1s and high in most cDC2s (Fig. 4e). However, although single pre-cDC1 cells have relatively higher expression of *IRF8* and relatively lower expression of *IRF4* than those in pre-cDC2 subpopulation (Fig. 4e), neither *IRF8* nor *IRF4* expression is mutually exclusive between two pre-cDC subpopulations. In particular, *IRF8* has a bimodal expression pattern in pre-cDCs (lower in pre-cDC2 cells and higher in pre-cDC1 cells) as opposed to its unimodal expression (higher in cDC1 and lower in cDC2) in cDCs as shown in Fig. 4f. Several single pre-cDC2 cells express *IRF8*, which seemingly contradicts their fates to cDC2. On the other hand, we observe that *IRF8/IRF4* ratio is unimodal in pre-DC1 and pre-DC2, i.e., pre-cDC2 cells exhibit lower *IRF8/IRF4* ratio compared to that of pre-cDC1. In addition, *IRF8/IRF4* ratio is consistently higher in pre-cDC1s than that of pre-cDC2s (*P* = 4.8e-6, Wilcoxon rank sum test, Fig. 4f). Therefore, the expression ratio of *IRF8/IRF4,* not the individual TF expression*,* correlates with pre-cDC’s commitment to respective cDC subset.

To confirm the ability of MR TFs in defining pre-committed pre-cDC populations, we then applied our method to examine three recently published scRNA-Seq datasets of peripheral blood cDC1, cDC2 and pre-cDCs from Breton et al. [5], Villani et al. [7] and See et al. [6]. (Additional file 1: Figure S8). For the dataset from Breton et al., these TFs can also separate cDC1 and cDC2, with pre-cDCs also showing pre-commitment pattern to either type of cDCs from with cluster C1 and C2 containing cDC2s and cDC2 pre-committed pre-cDCs and cluster C3 containing cDC1 and cDC1 pre-committed pre-cDCS. Additionally, the expression ratio of *IRF8/IRF4* correlates with pre-cDC’s commitment to respective cDC subset rather than expression level of *IRF8* or *IRF4* alone (Additional file 1: Figure S8A). CD172a(SIRPA) is a surface marker that can distinguish the two pre-cDC subsets, with higher expression indicating pre-commitment to cDC2 and lower expression indicating pre-commitment to cDC1 [5]. Consistently, the pre-cDCs in cluster C1 and cluster C2(i.e., pre-DC2) contain more cells expressing CD172a than that of cluster 3(i.e., pre-DC1). Similar observation was identified for the dataset from Villani et al. and See et al. [6, 7]) (Additional file 1: Figure S8B-C). This supports that our list of MR TFs can separate single pre-cDCs into pre-committed pre-cDC1 and pre-cDC2.

### Transcriptional signature of the two pre-cDC populations

**Fig. 5:**
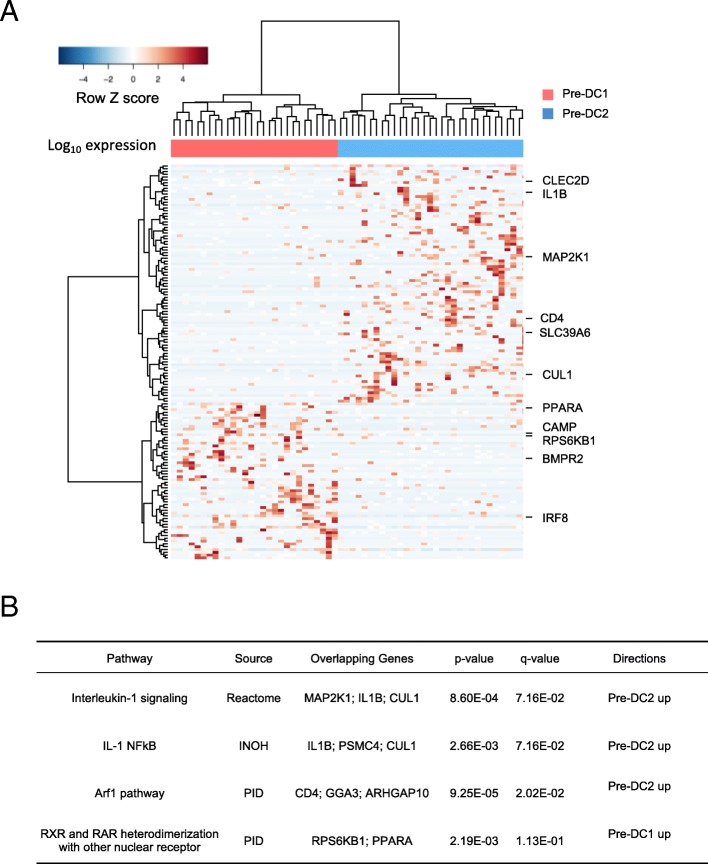
Characterization of the pre-cDC subpopulations. **a** Heatmap showing genes that are differentially expressed between the two subpopulations of pre-cDCs. **b** Enriched pathways with genes upregulated in each pre-cDC subset

To further characterize the pre-cDC1s and pre-cDC2s, we used SCDE [21] to derive the differentially expressed genes between the two subpopulations of single cells (*p*-value < 0.05, Additional file 2: Table S6 and Fig. 5a) and then performed pathway enrichment analysis with CPBD [22]. Genes upregulated in the pre-cDC1 subpopulation were enriched in RXR and RAR heterodimerization with other nuclear receptor pathway. Genes upregulated in the pre-cDC2 subpopulation were enriched in Interleukin-1 related pathways (Fig. 5b).

## Discussion

Previous studies [5, 6, 9] showed human pre-cDC cells are heterogeneous and contain pre-committed subpopulations that correspond to the two major cDC subtypes. However, the key molecular drivers of pre-commitment in human pre-cDCs were not investigated. To address this question, we performed single cell and bulk RNA-Seq of two cDC subsets and pre-cDCs from human peripheral blood and developed a new approach that could identify the underlying molecular recipe for pre-cDCs pre-commitment to terminal cDCs. Specifically, we performed single cell RNA-sequencing analysis by mixing different cell types in the experimental design to demonstrate that global gene expression pattern can distinguish pre-cDCs from cDCs, and separate the two conventional cDC subsets (cDC1 and cDC2) independent of surface markers. We found that neither highly variable genes within pre-cDCs, nor differentially expressed genes between cDC1 and cDC2 could separate the pre-DC subsets. We reasoned that it is possible that the transcriptional program driving the pre-commitment has not been fully manifested in pre-cDC. Instead, we identified 16 candidate master regulators by searching for transcriptional factors: (a) that are differentially expressed between two cDC cell types, or (b) whose targets are enriched among differentially expressed genes between two cDC cell types. We showed that these candidate master regulators were able to separate pre-cDCs into two subpopulations, resembling cDC1s and cDC2s, respectively. Furthermore, these two pre-cDC subpopulations are more correlated with the ratio of *IRF8* to *IRF4* expression than their individual expression level. This suggests combinatorial dose of transcription factors determines fate decision. Finally, we confirmed these findings using two recently published scRNA-Seq datasets [5, 7].

Using clustering analysis of single cells transcriptome data, we can identify pre-cDC, cDC1 and cDC2 from cell mixture across different experiment batches and conclude 1) global transcriptome of pre-cDCs are distinct from their immediate progeny cDCs and 2) cDCs single cells themselves are separable on the whole single cell transcriptome without prior knowledge of cell identity independent of cell surface markers. However, although the global transcriptome separated pre-cDC, cDC1 and cDC2, it could not separate the two pre-cDC subpopulations. This is consistent with recent studies that were trying to identify a critical subset of genes instead of the global transcriptome to reveal cell population heterogeneity [5, 6, 9, 28].

In the signature gene set analysis for the three populations, single pre-cDCs demonstrate more precursor features (signature genes were enriched for EGF-EGFR1 signaling and mitotic pathway) than cDCs and cDC population shows more mature characteristics in terms of immune function. cDC1 signature genes highlight its role in IFN-gamma functional pathway; cDC1s not only induce Th1 cells to produce high concentrations of interferon-gamma [29], but also respond to IFN-gamma, thereby serving as key regulator or perpetuator of Th1 response in vivo. cDC2 signature genes are enriched for pathways such as TGF beta signaling and toll-like receptor signaling which also corresponds to previous finding [30, 31].

The finding that biologically variable genes in the pre-cDC population can separate the two cDCs subsets implies that although pre-cDCs are functionally immature in comparison to cDCs, the biologically variable genes in pre-cDC population include components that can distinguish transcriptional program of cDC1 and cDC2, indicating the transcriptional program towards the differentiation into cDC1 or cDC2 is already initiated in pre-cDCs. However, using the full set of differentially expressed genes between cDC1 and cDC2, we were not able to separate the pre-committed pre-cDC subpopulations. We hypothesize that the commitment transcriptional program may not be fully implemented, therefore, we should examine whether the master regulators drive the differentiation process.

With a list of inferred candidate MR TFs, we were able to identify two pre-cDC subpopulations with each being close to one of the cDC types. Pre-cDC2(close to cDC2) cells have upregulated pathways related with Interleukin-1 which is consistent with the cDC2 specific signature gene set enriched pathway analysis (IL1/4/5 signaling pathways were enriched); and pre-cDC1 (close to cDC1) cells have upregulated pathways related with RXR and RAR pathway, which was previously shown to promote DC antigen presentation, differentiation and survival [32]. RXR and RAR are also well known to directly bind to *AP-1*, which is an essential protein to form a complex with *IRF8* and *BATF3* [33, 34]. Our findings suggest a new role of retinoic acid in cDC1 development. The pathway analysis also suggests that pre-cDC2 cells are more transcriptionally mature and express more cytokines than those (i.e., pre-cDC1) that pre-commit to cDC1 which may still need to undergo more development and differentiation steps towards to cDC1 stage.

Of these candidate MR TFs, functions of IRF8 have been studied in dendritic cells. Mutations in IRF8 could result in a complete lack of circulating dendritic cells or selective depletion of CD1c + circulating dendritic cells(cDC2s) [35]. Maintaining of IRF8 high expression level is required for cDC1 identity [10]. It is also found that IRF8 is strictly required for the survival of cDC1s [36]. Interestingly, CEBPB forms a negative feedback loop with IRF8 when specifying monocyte-derived DC and pDC chromatin states [37]. Consistently, they also show opposite expression direction in our human cDC1s and cDC2s (Fig. 4c). CEBPD belongs to the same family with CEBPB and can regulate IRF4 and IRF8 (Fig. 4c), which may also play a role in cDCs differentiation.

The t-SNE analysis with MR TFs indicates the combinatorial dose of multiple TFs is a likely to orchestrate the lineage program. This is consistent with the dosage-dependent functions of TFs including *IRF4* and *IRF8*. In particular, both *IRF4* and *IRF8*, due to their low affinity to interferon responsive element, must be recruited by other TFs to the DNA, and their function critically depends on forming protein-protein complexes [34]. Our result is consistent with a model in which competition between the two transcriptional factors *IRF8* and *IRF4* contributes most to the fate choices to cDC1 and cDC2 lineages. This is in line with the antagonism between *IRF4* and *IRF8* in the activated B cell differentiation into plasmablasts or undergoing affinity maturation in germinal centers [38]. Future experiments, such as ATAC-Seq [39] for profiling chromatin states, should test the effect of IRF4 and IRF8 dose changes on pre-DC1 and pre-DC2 lineage determination.

In our experimental design, instead of sequencing each single cell type separately in each batch, we mixed two cDC subsets in the first batch and pre-cDCs and two cDC subsets in the third batch, respectively. This strategy can help bypass batch effect as a potential confounding factor when comparing different populations. With the rapid development of single cell sequencing techniques, sequencing large scale of cells together is becoming feasible: the Fluidigm C1 mRNA Seq HT IFC can capture up to 800 cells in a run [40], microfluidic system can sequence scalable number of cells at low cost [41] and Drop-seq can sequence thousands of cells each time [42].

## Conclusions

In this study, we performed single cell RNA-Seq to study the differentiation process of pre-cDCs. We found that pre-cDCs subpopulations can be identified based on expression of a small number of core transcriptional factors. Additionally, the combinatorial dose of *IRF4* and *IRF8* had a stronger association with the fate decision than their individual expression level. This study suggests the concept that combinatorial dose of transcription factors determines cell differentiation fate.

## Methods

### Single cell capture and library preparation for RNA sequencing

#### Single cell isolation

Human peripheral blood samples were purchased from New York Blood Center (New York) and processed 24-48 h post-collection. Fresh mononuclear cells were isolated from cord blood or peripheral blood by density centrifugation using Ficoll-Hypaque (Amersham Pharmacia Biotech, Piscataway, NJ).

For isolation of cDC1, cDC2, and rare pre-cDCs from peripheral blood, an enrichment step was also performed prior to FACS sorting. In brief, mononuclear cells were incubated with antibodies against CD135 (4G8, PE, BD) and CD117 (A3C6E2, Biotin, Biolegend) for 40 min at 4 °C. After washing, antibody against PE (PE001, Biotin, Biolegend) was added and incubated for another 10 min at 4 °C. Following wash, CD117+ and CD135+ cells were positively selected using anti-biotin MicroBeads and LS MACS magnetic columns (Miltenyi). For sorting pre-cDCs, enriched cells were stained for CD14 (TuK4, Qdot-655, Invitrogen), CD3 (OKT3, Brilliant Violet (BV) 650, Biolegend), CD19 (HIB19, BV650, Biolegend), CD56 (HCD56, BV650, Biolegend), CD66b (G10F5, PerCP-Cy5.5, Biolegend), CD303 (201A, PerCP-Cy5.5, Biolegend), CD1c (L161, APC-Cy7, Biolegend), CD141 (M80, PE-Cy7, Biolegend), CD34 (581, AlexaFluor700, Biolegend), CD117 (104D2, BV421, Biolegend), CD135 (4G8, PE, BD), CD45RA (HI100, BV510, Biolegend), CD116 (4H1, FITC, Biolegend) and CD115 (9-4D2-1E4, APC, Biolegend) for 40 min on ice. Pre-cDCs were isolated as Lin(CD3/19/56/14)- Granulocyte(CD66b)-pDC(CD303)-cDC(CD1c/CD141)-CD34-CD117 + CD135 + SSC lo CD116 + CD115-CD45RA+ cells. cDC1 were isolated as Lin(CD3/19/56/14)- Granulocyte(CD66b)-pDC(CD303)-CD141+ cells. cDC2 were isolated as Lin(CD3/19/56/14)- Granulocyte(CD66b)-pDC(CD303)-CD1c + cells.

Collected cells were washed and resuspended in 0.1% BSA (Fisher), 2 mM EDTA, PBS. Purified populations were then mixed at specific ratios for different sequencing batches. For batch 1, cDC1 and cDC2 were mixed at a 1:1 ratio; for batch 2, pre-cDCs were not mixed; for batch 3, pre-cDCs, cDC1 and cDC2 were mixed at a 2:1:1 ratio, respectively. All mixes had a final cellular concentration of 200 total cells/ul and 5ul of sample mix was loaded on Fluidigm C1.

### Library preparation for RNA sequencing

#### Fluidigm C1

Fluidigm C1 was run according to manufacturer’s instructions, including using the LIVE/DEAD stain (L-3224, LifeTechnologies). They were imaged using a fluorescent microscope. However, ERCC ExFold RNA-Spike-Ins (4,456,739, Ambion) were used in place of the RNA spikes as recommended. For all the three experiments, the overall spike-in dilution was 1: 2, 000, 000.

#### Sequencing

cDNA was quantified using the High Sensitivity Qubit kit (Q32854, LifeTechnologies) and EnVision plate reader. All experiments were performed using an Illumina NextSeq500 with 75 bp single-end reads and ~ 400 M reads/run.

### Samples and library preparation for bulk RNA sequencing

Blood samples were obtained in the same way with that of single cells. For isolation of cDC1, cDC2, and rare pre-cDCs from peripheral blood, the process is the same with that of single cells.

Bulk sequencing libraries were created using the SMARTer Ultra Low RNA Kit (634,935 and 639,207, Clontech) and sequenced with an Illumina NextSeq500 with 75 bp single-end reads and ~ 400 M reads/run).

### Computational analysis

Most of the computational analysis was performed in the R programming environment, unless stated otherwise. Multi-dimensional scaling was performed with cmdscale() function with eig = TRUE and k = 2. Heatmap was done with heatmap.2() in gplots package. Principal components analysis was performed with the function *prcomp()*, with centering, scaling and cor options on. Identification of the upstream regulators of a gene set was done with Enrichr [43] using the ChEA database (version ChEA 2016). Pathway analysis was done with ConsensusPathDB [22], which is a meta-database that integrates 4593 pathways from different sources. Signature analysis to assess the purity of cDC clustering were performed in two ways: 1) the connectivity map score [44], which measures the closeness of each single cell to a set of cell type signature genes, calculated with the connectivity_score() function of gmap package [45]; 2) weighted summation score: $\sum \limits_{i=1}^n{w}_i{x}_i$, where the weight *w*_*i*_ for each gene *i* is the fold change between cDC1 VS. cDC2 bulk samples and the expression value for each gene *i* is binary: 0 if the gene is not expressed; 1 if the gene is expressed. Rand index, which measures the agreement between two clustering results, was calculated with adjustedRand() function of clues package [46].

### Single-cell RNA sequencing data analysis

For all three batches of cells, we mapped single-end 75 bp reads to the human reference genome (Ensembl GRCh37) and the ERCC sequences using STAR [47] (version 2.3.0e) with default parameters. We used featureCounts [48] to compute the number of reads mapped to each gene or spike-in with options “-s 0 -t exon -g gene_name” . Only uniquely mapped reads were considered here in downstream analysis. We excluded cells that have fewer than 100,000 reads from the downstream analysis. We used the SCDE package [21] to fit individual error models for each cell and calculated an adjusted distance measure between each pair of individual cells that accounted for the probability that genes were not observed because of amplification failures or other reasons, i.e., dropout issue. We then used this adjusted distance measure as input to a multi-dimensional scaling (MDS) analysis with 2 dimensions for visualization and clustering of individual cells.

### Bulk RNA sequencing data analysis

We sequenced 5 pre-cDCs samples, 4 cDC1 samples and 4 cDC2 samples. Stranded single-end 75 bp sequenced with NextSeq reads were mapped to the human reference genome (Ensembl GRCh37) using STAR [47] (version 2.3.0e) with default parameters. We used RNA-SeQC [49] and RSeQC [50] to do quality control for the aligned reads. Samples that have high intergenic rate and reverse strand rate of mapped reads were removed. Finally, we have 3 good samples for each type of cells. featureCounts [48] was also used to obtain the number of uniquely mapped reads in sense direction to each gene with options “-s 1 -t exon -g gene_name”. The raw data of both single cell samples and bulk samples was uploaded to NCBI GEO (accession number GSE81682).

### Inference of single cell populations signature gene set

The read counts were first normalized with DESeq2 [51] to remove the sequencing depth difference and then log2 transformed with pseudo count 1 added. The list of genes that are differentially expressed between pre-cDCs, cDC1 and cDC2 was identified with one-way analysis of variance aov() function in R. TukeyHSD() function was used to do Tukey HSD post-hoc test and Benjanmini-Hochberg multiple testing correction. Four hundred sixty-seven genes were identified with absolute value of log_2_ fold change larger than one and adjusted *p*-value < 0.05. Unsupervised clustering of the 467 genes and 135 single cells were done with Ward agglomeration method and distance was defined as 1- Spearman correlation. The predicted regulator TFs here were selected by: a) p.adj < 0.05; b); log2FC > 2; c) combined score > 0; d) expressed in at least 10 cells in the corresponding cell population.

### Highly variable genes in pre-cDCs inference

To determine genes with high biological variability in pre-cDCs, we first used the method introduced by Brennecke et al. [52] to fit the dependence between the mean of normalized read counts and the squared coefficient of variation (CV^2^) with ${CV}^2=\frac{a_1}{\mu }+{\alpha}_0$ on 92 spike-ins for pre-cDCs in batch 2 only, where μ is the mean of normalized read counts, *a*_1_ and *α*_0_ are coefficients obtained from the fit. Then, for each gene, we use the fitted technical estimate and test against the null hypothesis that the biological coefficient of variation is less than 50% (at 10% FDR). This leads to 1389 variable genes. Among these genes, 842 genes were detected in at least in 10 cells and were selected as biological variable genes to perform MDS for all the single cells.

### Pre-cDCs pre-committed subpopulation visualization

To identify the differentiation map from pre-cDCs to cDCs, we first inferred a list of candidate master regulator [15] TFs that are significantly differentially expressed between bulk cDC1 and cDC2 (BH p.adj < 0.05, two-fold change) or have regulated targets enriched in the differentially expressed genes and exhibit marginal differential expression between bulk cDC1 and cDC2 (pvalue < 0.05, two-fold change). Then we define the distance between cell *x* and *y* as 1- r(x, y), where r represents Spearman’s rank correlation coefficient between x and y based on the expression level of MR TFs. At last, we use this distance matrix as input to t-Distributed Stochastic Neighbor Embedding (t-SNE) [53] implemented in R for visualization of the single cells with parameters perplexity = 20 and theta = 0.1.

## Additional files

Additional file 1 **Figure S1.** Flow chart of the analysis. **Figure S2.** Comparison of 3 studies: See et al., 2017, Villani et al., 2017 and Ma et al., 2018. **Figure S3.** Quality control of single cell sequencing data. (A) Sequencing saturation analysis for the 3 batches. (B) Bar plot of metrics to assess sequencing quality for all the single cells. **Figure S4.** Outlier analysis. (A) Multidimensional scaling (MDS) plot indicates that group 3 is outliers (dots outside of the dashed ovals). (B) Boxplot of mapped reads number for good single cells (groups1 and 2) and outliers (group 3). (C) Histogram of the percentage of mitochondrial reads, genes detected out of 100 housekeeping genesand 496 cell cycle genes. (D) Heatmap of cell-specific markers for pre-cDC and cDCs. **Figure S5.** Assessment of the purity of the two DC clusters. Cmap score for each single cell using DC signature genes from Villani et al. (A) and signature genes from our bulk RNA-Seq data (B). (C) histogram of weighted sum score with the signature genes from our bulk RNA-Seq data.**Figure S6.** More details about MR TFs between bulk cDC1 and cDC2 that potentially drive the pre-commitment of pre-DCs. (A-B) Heatmap of MR TFs in bulk data (A) and single cell data (B). (C) t-SNE plot of all the single cells with global transcriptome, biological variable genes in pre-cDCs, DE genes between bulk cDC1 and cDC2 and the MR TFs, with pre-committed pre-cDC subsets marked. (D) Violin plot of the expression for the housing keep gene GABARAP. **Figure S7.** Trajectory analysis with Monocle2. **Figure S8.** Test our hypothesis on three published data sets. Test our hypothesis on the dataset of Breton et al., [5](A), Villani et al., [7](B) and the dataset in Fig. 3 of See et al., 6(C). (PPTX 5054 kb)

Additional file 2: **Table S1.** The list of 380 genes that are differentially expressed between at least one pair of cell populations in mean expression and the gene clustering result. **Table S2.** A) The list of biological variable genes in pre-cDC of batch 2, B) enriched pathways of the variable genes and C) upstream regulators of the variable genes. **Table S3.** The list of random selected genes to generate MDS plot in Fig. 3d. **Table S4.** The list of cell cycle genes from reactome to generate MDS plot in Fig. 3e. **Table S5.** Summary of master regulator transcriptional factors. Their expression level comparison was shown in groups bulk cDC2 VS. cDC1, single cell cDC2 VS. cDC1 and single cell pre-DC2 VS. pre-DC1. For the TFs that have targets enriched in the differentially expressed genes between cDC1 and cDC2, the evidence from ChEA database (version 2016) was followed. **Table S6.** The list of differentially expressed genes between two pre-cDC subpopulations. (ZIP 288 kb)

## Data Availability

The accession number for the RNA-Seq data reported in this paper is GEO: GSE89322.

## Abbreviations

cDC1: cD141+ cDC
cDC2: cD1c + cDC
cDCs: classic dendritic cells
MDS: Multidimensional scaling
MR TF: Master regulator transcriptional factors
scRNA-Seq: single cell RNA sequencing
t-SNE: t-Distributed Stochastic Neighbor Embedding
