# Supplementary material for: Single cell RNA-Seq reveals pre-cDCs fate determined by transcription factor combinatorial dose
Source: BMC Mol Cell Biol. 2019 Jun 28;20:20. doi: 10.1186/s12860-019-0199-y (PMC6599345; doi:10.1186/s12860-019-0199-y)
Supplement: Supplementary file 1 — Figure S1. Flow chart of the analysis. Figure S2. Comparison of 3 studies: See et al., 2017, Villani et al., 2017 and Ma et al., 2018. Figure S3. Quality control of single cell sequencing data. (A) Sequencing saturation analysis for the 3 batches. (B) Bar plot of metrics to assess sequencing quality for all the single cells. Figure S4. Outlier analysis. (A) Multidimensional scaling (MDS) plot indicates that group 3 is outliers (dots outside of the dashed ovals). (B) Boxplot of mapped reads number for good single cells (groups1 and 2) and outliers (group 3). (C) Histogram of the percentage of mitochondrial reads, genes detected out of 100 housekeeping genesand 496 cell cycle genes. (D) Heatmap of cell-specific markers for pre-cDC and cDCs. Figure S5. Assessment of the purity of the two DC clusters. Cmap score for each single cell using DC signature genes from Villani et al. (A) and signature genes from our bulk RNA-Seq data (B). (C) histogram of weighted sum score with the signature genes from our bulk RNA-Seq data.Figure S6. More details about MR TFs between bulk cDC1 and cDC2 that potentially drive the pre-commitment of pre-DCs. (A-B) Heatmap of MR TFs in bulk data (A) and single cell data (B). (C) t-SNE plot of all the single cells with global transcriptome, biological variable genes in pre-cDCs, DE genes between bulk cDC1 and cDC2 and the MR TFs, with pre-committed pre-cDC subsets marked. (D) Violin plot of the expression for the housing keep gene GABARAP. Figure S7. Trajectory analysis with Monocle2. Figure S8. Test our hypothesis on three published data sets. Test our hypothesis on the dataset of Breton et al., [5](A), Villani et al., [7](B) and the dataset in Fig. 3 of See et al., 6(C). (PPTX 5054 kb) [file 12860_2019_199_MOESM1_ESM.pptx]

## Slide 1
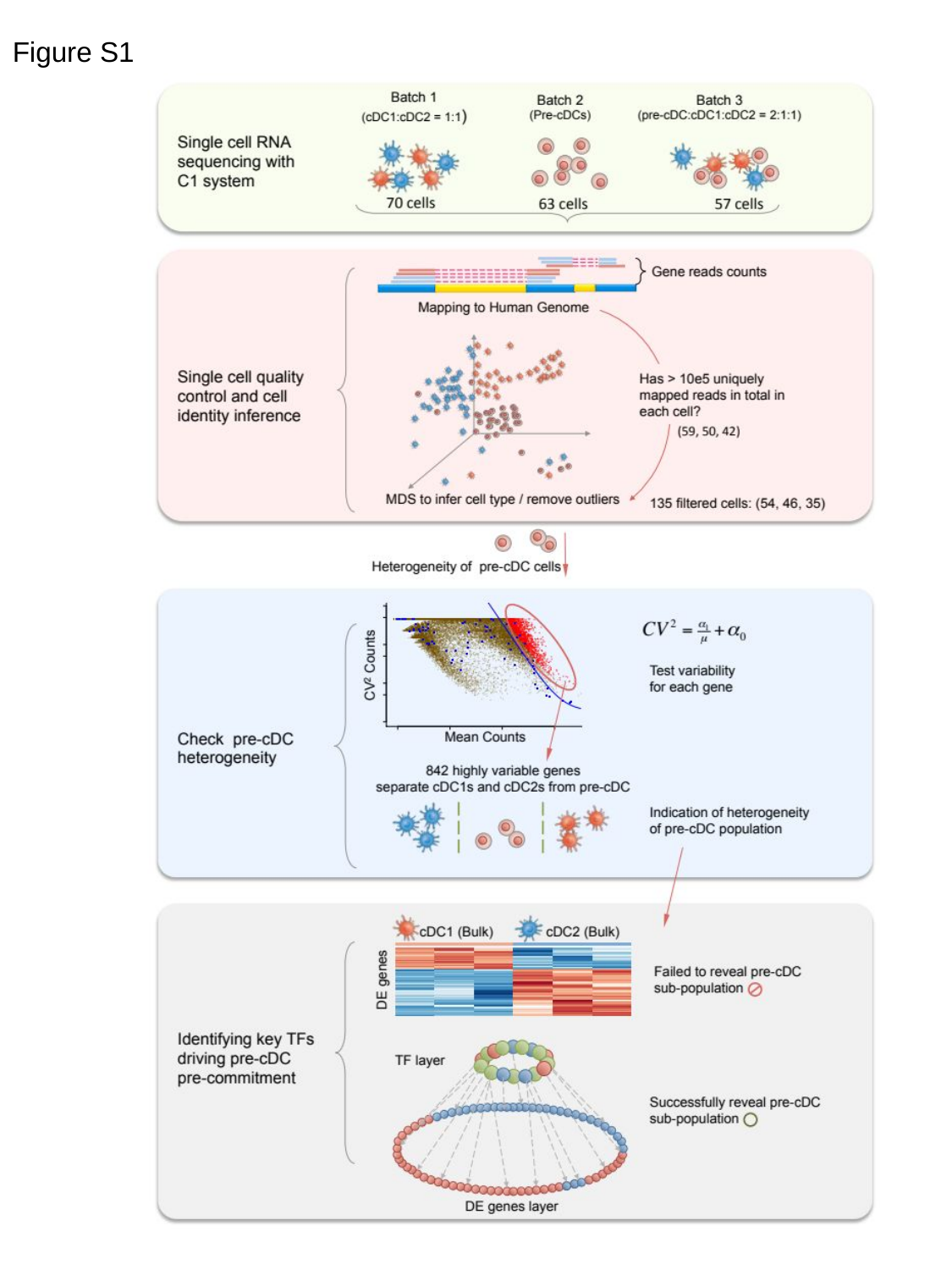

Figure S1

## Slide 2
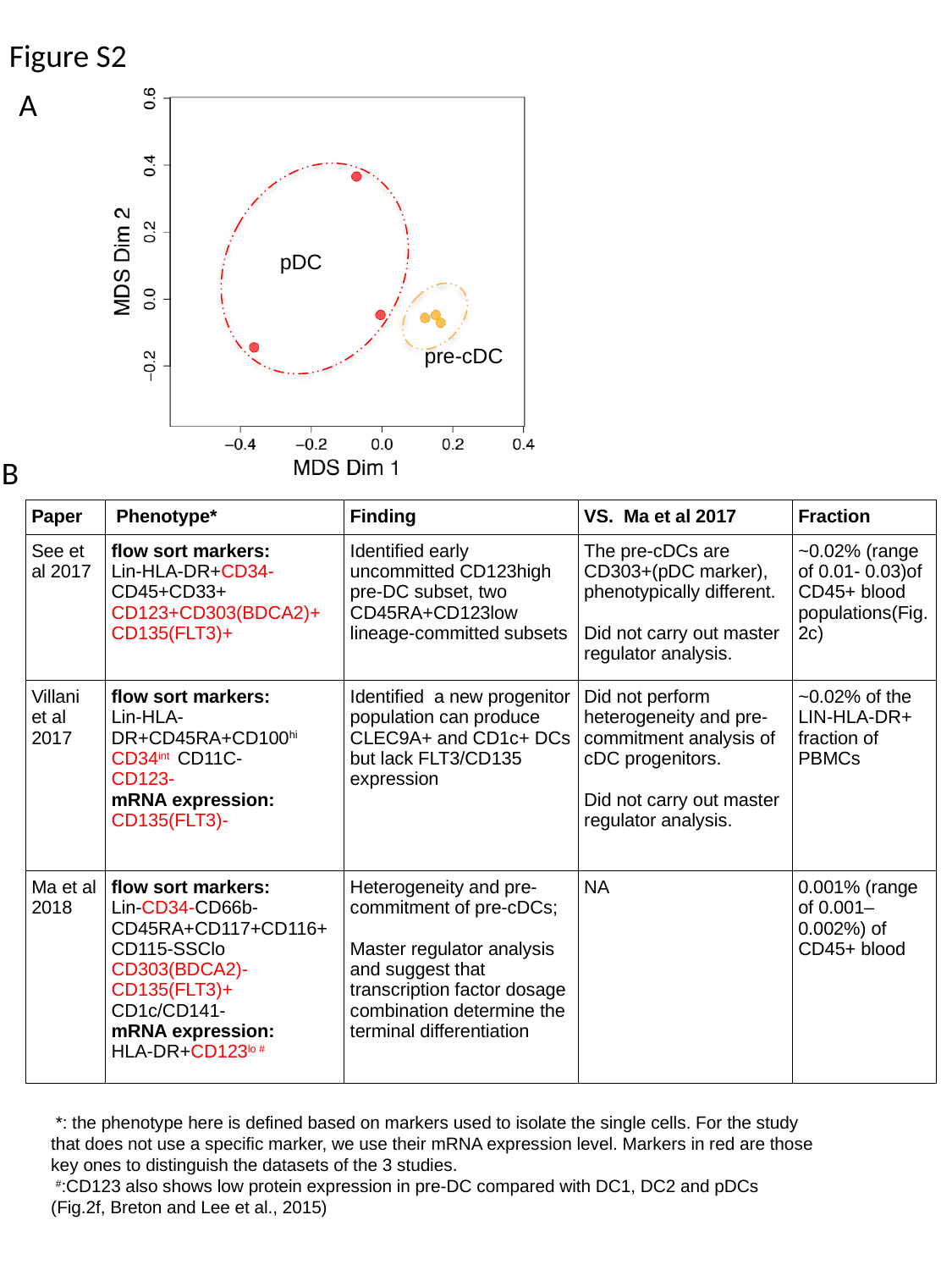

Figure S2
A
pDC
pre-cDC
B
| Paper | Phenotype\* | Finding | VS. Ma et al 2017 | Fraction |
| --- | --- | --- | --- | --- |
| See et al 2017 | flow sort markers: Lin-HLA-DR+CD34-CD45+CD33+ CD123+CD303(BDCA2)+ CD135(FLT3)+ | Identified early uncommitted CD123high pre-DC subset, two CD45RA+CD123low lineage-committed subsets | The pre-cDCs are CD303+(pDC marker), phenotypically different. Did not carry out master regulator analysis. | ~0.02% (range of 0.01- 0.03)of CD45+ blood populations(Fig.2c) |
| Villani et al 2017 | flow sort markers: Lin-HLA-DR+CD45RA+CD100hi CD34int CD11C- CD123- mRNA expression: CD135(FLT3)- | Identified a new progenitor population can produce CLEC9A+ and CD1c+ DCs but lack FLT3/CD135 expression | Did not perform heterogeneity and pre-commitment analysis of cDC progenitors. Did not carry out master regulator analysis. | ~0.02% of the LIN-HLA-DR+ fraction of PBMCs |
| Ma et al 2018 | flow sort markers: Lin-CD34-CD66b- CD45RA+CD117+CD116+CD115-SSClo CD303(BDCA2)-CD135(FLT3)+ CD1c/CD141- mRNA expression: HLA-DR+CD123lo # | Heterogeneity and pre-commitment of pre-cDCs; Master regulator analysis and suggest that transcription factor dosage combination determine the terminal differentiation | NA | 0.001% (range of 0.001– 0.002%) of CD45+ blood |
 *: the phenotype here is defined based on markers used to isolate the single cells. For the study that does not use a specific marker, we use their mRNA expression level. Markers in red are those key ones to distinguish the datasets of the 3 studies.
 #:CD123 also shows low protein expression in pre-DC compared with DC1, DC2 and pDCs (Fig.2f, Breton and Lee et al., 2015)

## Slide 3
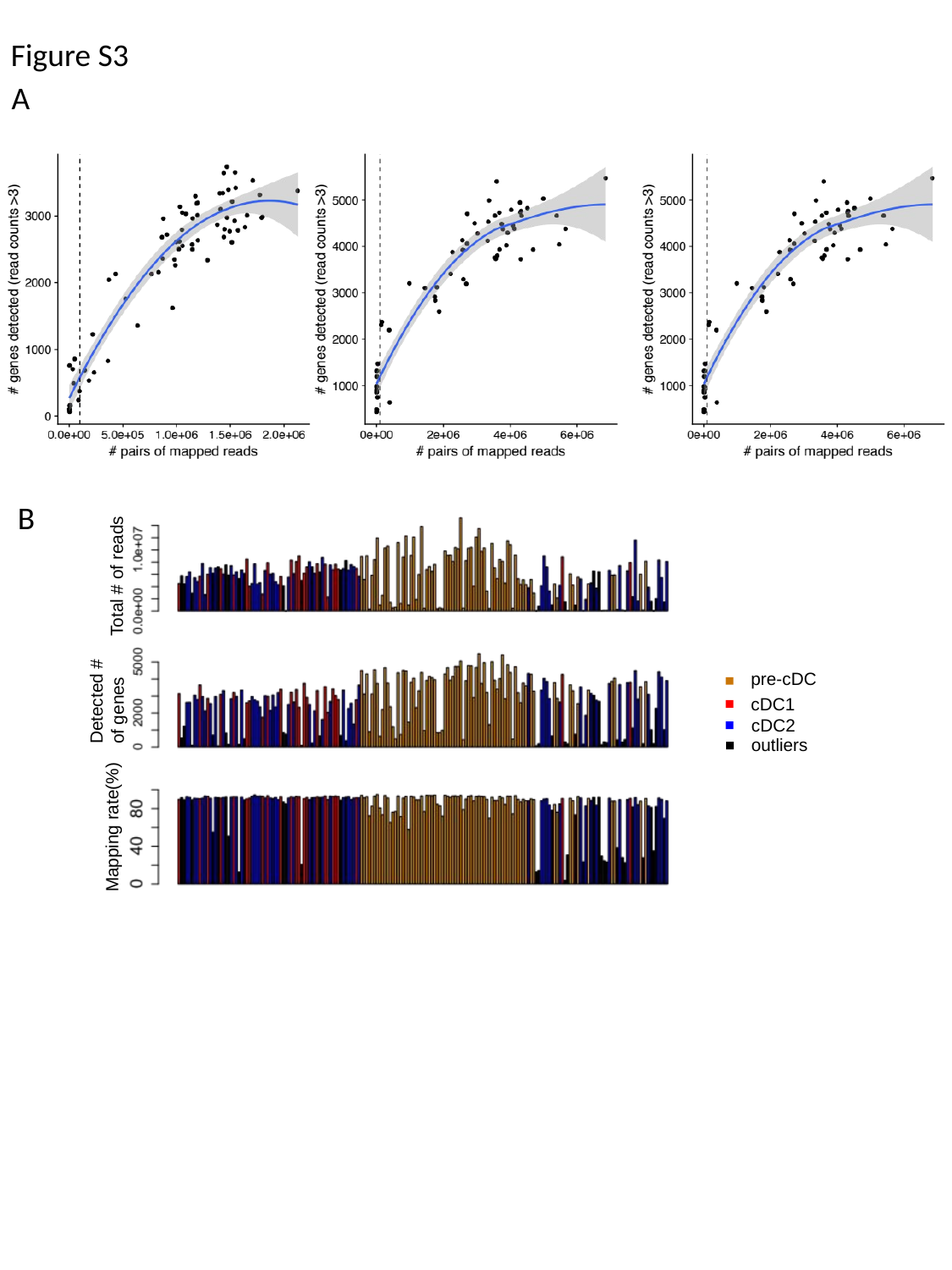

Figure S3
A
B
Total # of reads
pre-cDC
Detected # of genes
cDC1
cDC2
outliers
Mapping rate(%)

## Slide 4
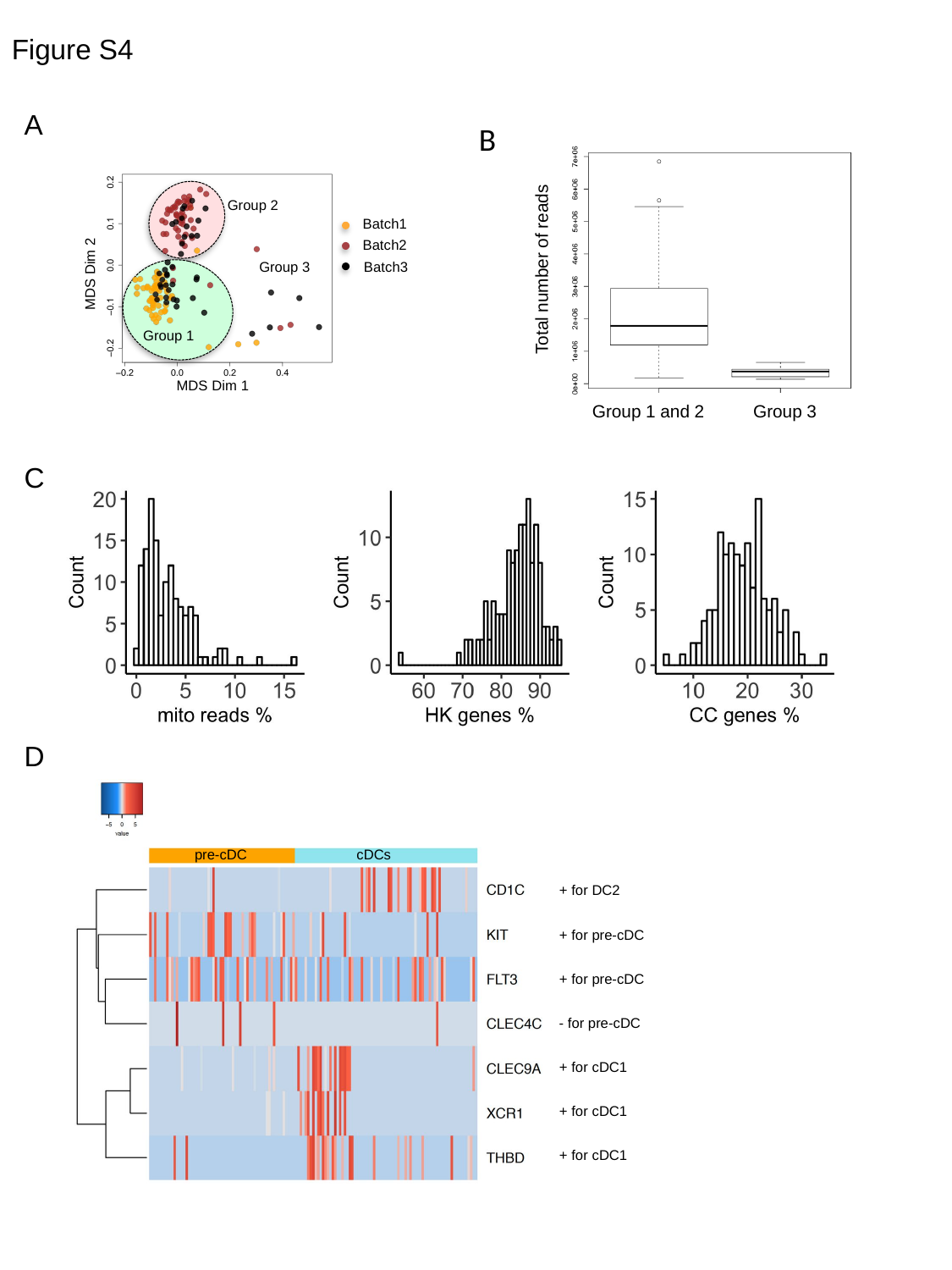

Figure S4
A
B
Total number of reads
Group 1 and 2
Group 3
Group 2
Batch1
Batch2
MDS Dim 2
Group 3
Batch3
Group 1
MDS Dim 1
C
D
pre-cDC
cDCs
+ for DC2
+ for pre-cDC
+ for pre-cDC
- for pre-cDC
+ for cDC1
+ for cDC1
+ for cDC1

## Slide 5
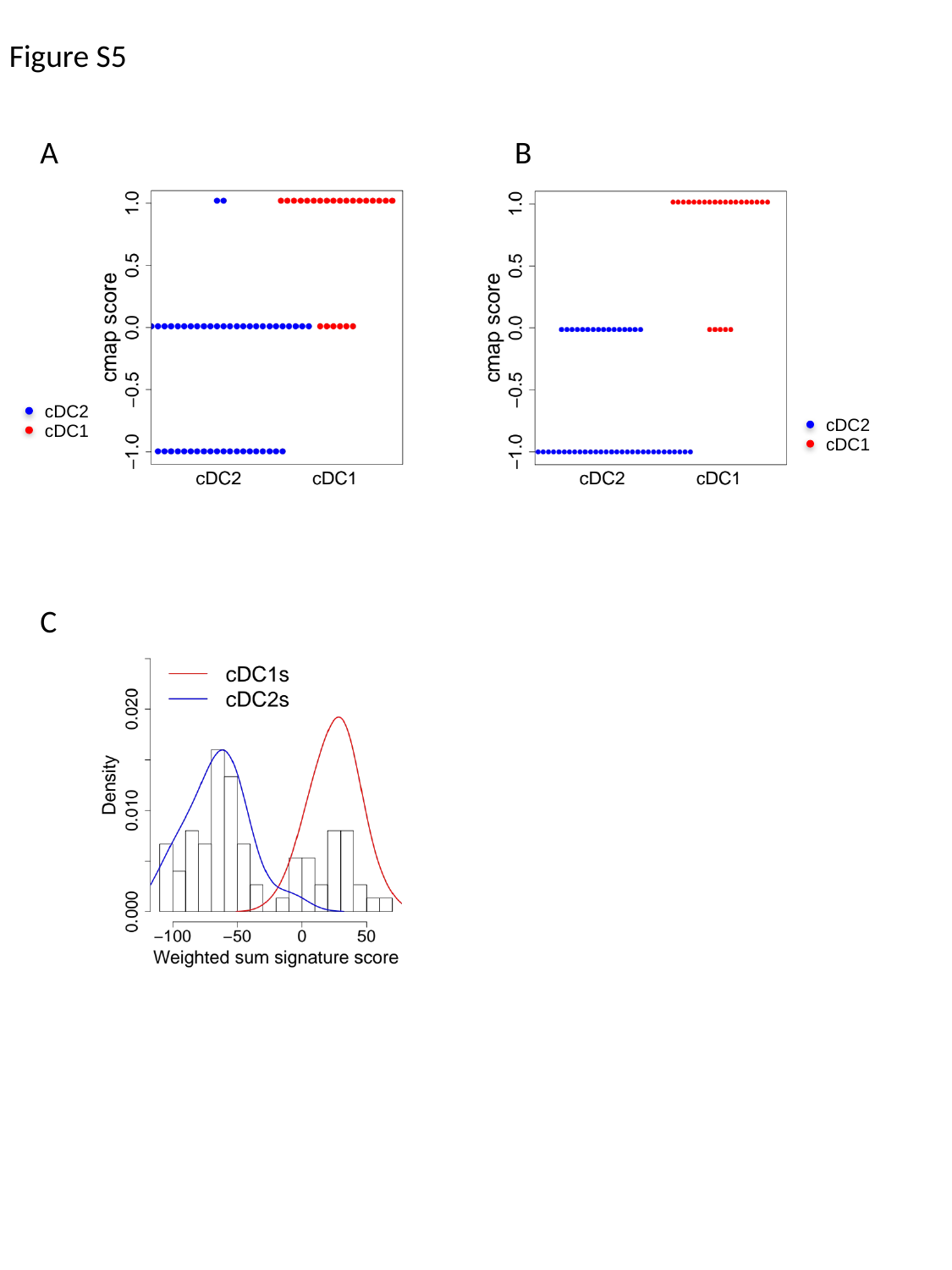

Figure S5
A
B
cDC2
cDC2
cDC1
cDC1
C

## Slide 6
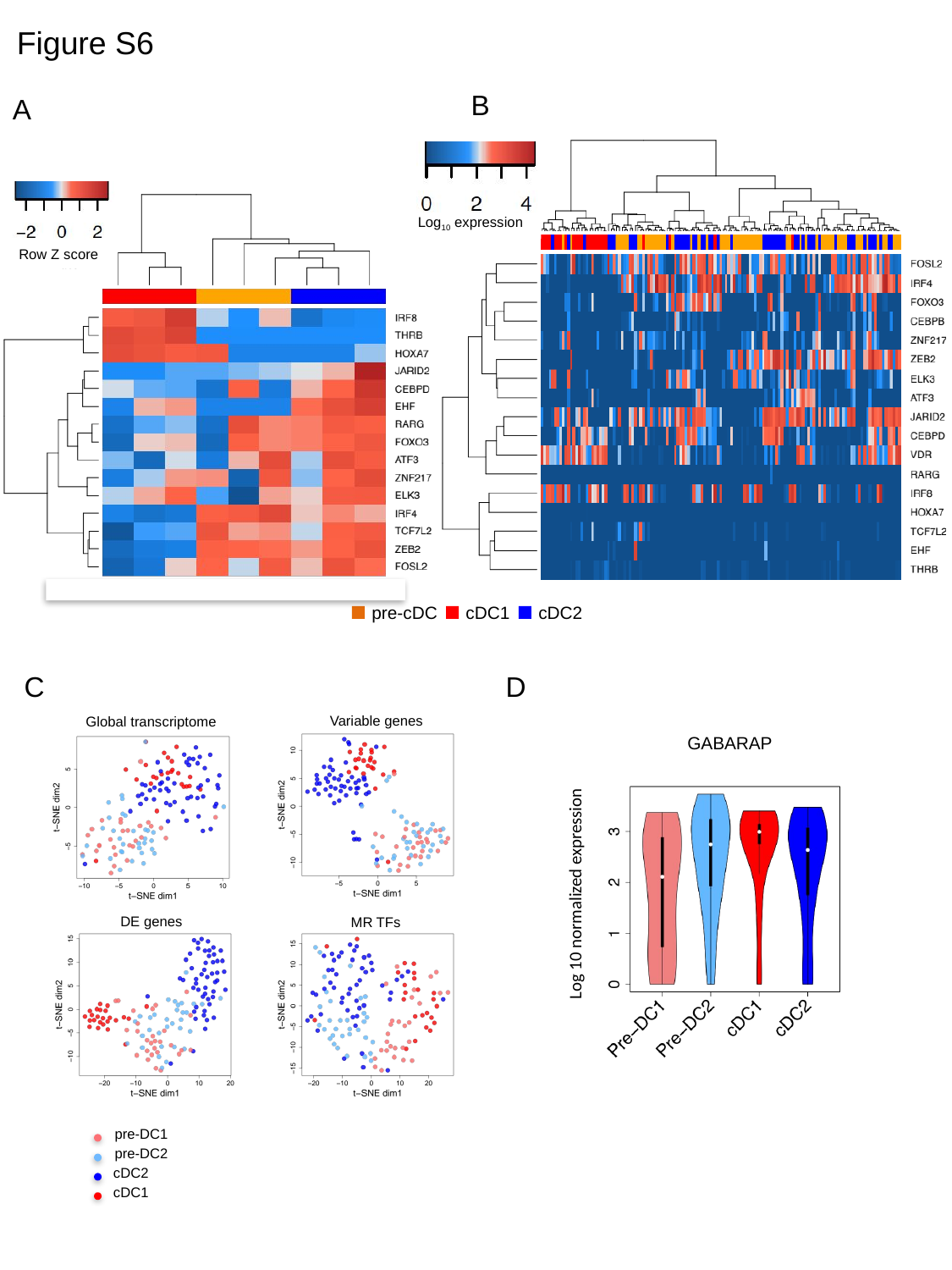

Figure S6
B
A
Log10 expression
Row Z score
pre-cDC
cDC1
cDC2
C
Variable genes
Global transcriptome
DE genes
MR TFs
pre-DC1
pre-DC2
cDC2
cDC1
D
GABARAP
Log 10 normalized expression

## Slide 7
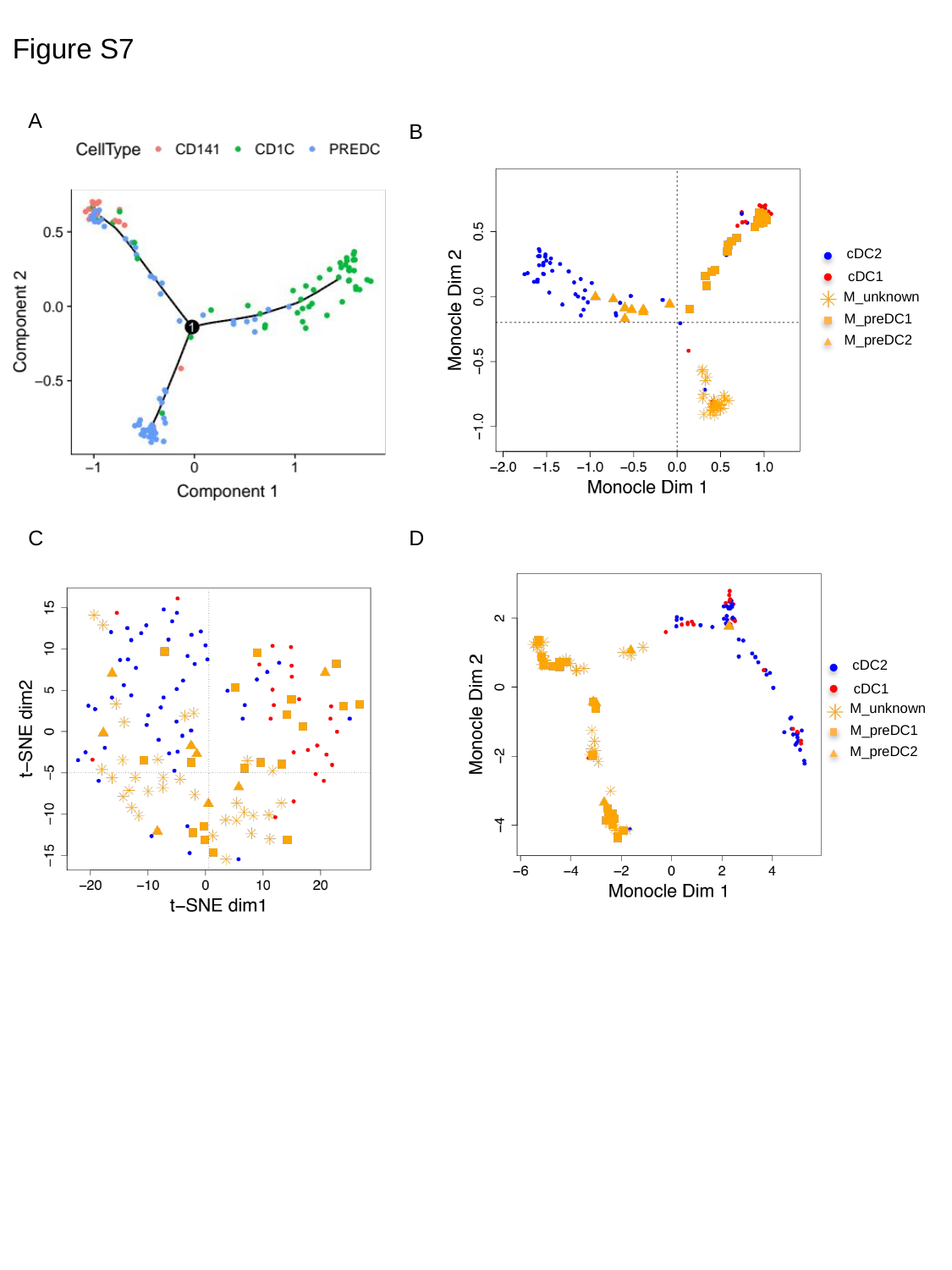

Figure S7
A
B
cDC2
cDC1
M_unknown
M_preDC1
M_preDC2
C
D
cDC2
cDC1
M_unknown
M_preDC1
M_preDC2

## Slide 8
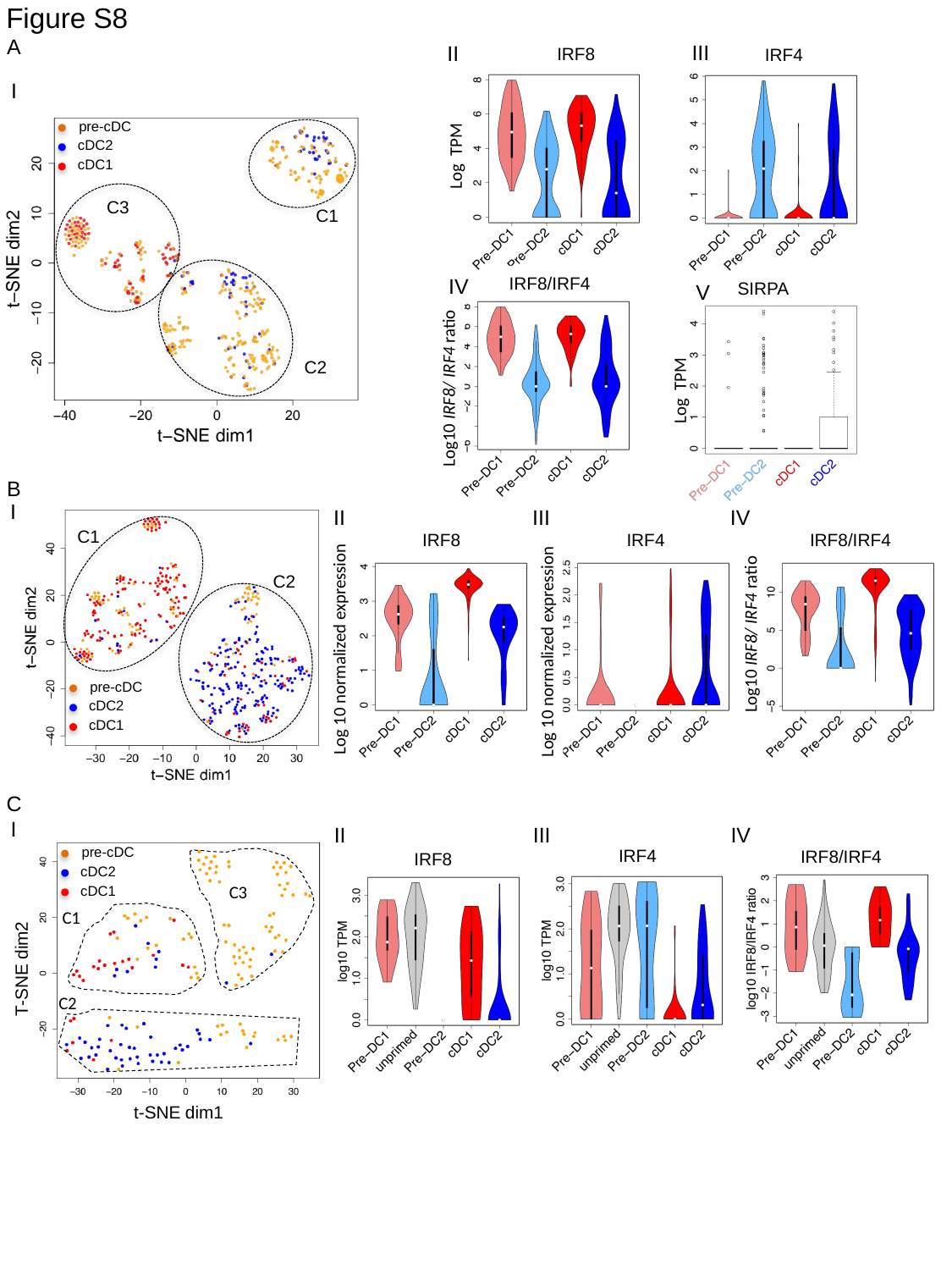

Figure S8
A
III
II
IRF8
IRF4
I
pre-cDC
cDC2
cDC1
Log TPM
C3
C1
IRF8/IRF4
IV
SIRPA
V
Log10 IRF8/ IRF4 ratio
C2
Log TPM
B
I
II
III
IV
C1
IRF8
IRF4
IRF8/IRF4
C2
Log10 IRF8/ IRF4 ratio
Log 10 normalized expression
Log 10 normalized expression
pre-cDC
cDC2
cDC1
C
I
II
III
IV
pre-cDC
cDC2
cDC1
IRF4
IRF8/IRF4
IRF8
C3
C1
T-SNE dim2
C2
t-SNE dim1
